# Biological and Clinical Implications of Gene-Expression Profiling in Diffuse Large B-Cell Lymphoma: A Proposal for a Targeted BLYM-777 Consortium Panel as Part of a Multilayered Analytical Approach

**DOI:** 10.3390/cancers14081857

**Published:** 2022-04-07

**Authors:** Fleur A. de Groot, Ruben A. L. de Groen, Anke van den Berg, Patty M. Jansen, King H. Lam, Pim G. N. J. Mutsaers, Carel J. M. van Noesel, Martine E. D. Chamuleau, Wendy B. C. Stevens, Jessica R. Plaça, Rogier Mous, Marie José Kersten, Marjolein M. W. van der Poel, Thomas Tousseyn, F. J. Sherida H. Woei-a-Jin, Arjan Diepstra, Marcel Nijland, Joost S. P. Vermaat

**Affiliations:** 1 Department of Hematology, Leiden University Medical Center, 2333 ZA Leiden, The Netherlands; f.a.de_groot@lumc.nl (F.A.d.G.); r.a.l.de_groen@lumc.nl (R.A.L.d.G.); 2 Department of Pathology, University Medical Center Groningen, University of Groningen, 9713 GZ Groningen, The Netherlands; a.van.den.berg01@umcg.nl (A.v.d.B.); jessicaplaca@usp.br (J.R.P.); a.diepstra@umcg.nl (A.D.); 3 Department of Pathology, Leiden University Medical Center, 2333 ZA Leiden, The Netherlands; p.m.jansen@lumc.nl; 4 Department of Pathology, Erasmus Medical Center, 3015 GD Rotterdam, The Netherlands; k.h.lam@erasmusmc.nl; 5 Department of Hematology, Erasmus Medical Center, 3015 GD Rotterdam, The Netherlands; p.mutsaers@erasmusmc.nl; 6 Department of Pathology, Amsterdam University Medical Center, 1105 AZ Amsterdam, The Netherlands; c.j.vannoesel@amsterdamumc.nl; 7 Cancer Center Amsterdam and LYMMCARE, Department of Hematology, Amsterdam University Medical Centers, 1105 AZ Amsterdam, The Netherlands; m.chamuleau@amsterdamumc.nl (M.E.D.C.); m.j.kersten@amsterdamumc.nl (M.J.K.); 8 Department of Hematology, Radboud University Medical Center, 6525 GA Nijmegen, The Netherlands; wendy.stevens@radboudumc.nl; 9 Department of Hematology, University Medical Center Utrecht, 3584 CX Utrecht, The Netherlands; r.mous@umcutrecht.nl; 10 Department of Internal Medicine, Division of Hematology, GROW School for Oncology and Developmental Biology, Maastricht University Medical Center, 6229 HX Maastricht, The Netherlands; marjolein.vander.poel@mumc.nl; 11 Department of Pathology, University Hospitals Leuven, 3000 Leuven, Belgium; thomas.tousseyn@uzleuven.be; 12 Department of General Medical Oncology, University Hospitals Leuven, 3000 Leuven, Belgium; sherida.woei-a-jin@uzleuven.be; 13 Department of Hematology, University Medical Center Groningen, University of Groningen, 9713 GZ Groningen, The Netherlands; m.nijland@umcg.nl

**Keywords:** gene-expression profiling, DLBCL, integration genomics, localization

## Abstract

**Simple Summary:**

This review summarizes gene-expression profiling insights into the background and origination of diffuse large B-cell lymphomas (DLBCL). To further unravel the molecular biology of these lymphomas, a consortium panel called BLYM-777 was designed including genes important for subtype classifications, genetic pathways, tumor-microenvironment, immune response and resistance to targeted therapies. This review proposes to combine this transcriptomic method with genomics, proteomics, and patient characteristics to facilitate diagnostic classification, prognostication, and the development of new targeted therapeutic strategies in DLBCL.

**Abstract:**

Gene-expression profiling (GEP) is used to study the molecular biology of lymphomas. Here, advancing insights from GEP studies in diffuse large B-cell lymphoma (DLBCL) lymphomagenesis are discussed. GEP studies elucidated subtypes based on cell-of-origin principles and profoundly changed the biological understanding of DLBCL with clinical relevance. Studies integrating GEP and next-generation DNA sequencing defined different molecular subtypes of DLBCL entities originating at specific anatomical localizations. With the emergence of high-throughput technologies, the tumor microenvironment (TME) has been recognized as a critical component in DLBCL pathogenesis. TME studies have characterized so-called “lymphoma microenvironments” and “ecotypes”. Despite gained insights, unexplained chemo-refractoriness in DLBCL remains. To further elucidate the complex biology of DLBCL, we propose a novel targeted GEP consortium panel, called BLYM-777. This knowledge-based biology-driven panel includes probes for 777 genes, covering many aspects regarding B-cell lymphomagenesis (f.e., MYC signature, TME, immune surveillance and resistance to CAR T-cell therapy). Regarding lymphomagenesis, upcoming DLBCL studies need to incorporate genomic and transcriptomic approaches with proteomic methods and correlate these multi-omics data with patient characteristics of well-defined and homogeneous cohorts. This multilayered methodology potentially enhances diagnostic classification of DLBCL subtypes, prognostication, and the development of novel targeted therapeutic strategies.

## 1. Introduction

The main challenge for diffuse large B-cell lymphoma (DLBCL), not otherwise specified (NOS), the most common lymphoid malignancy, is to improve survival outcomes. Approximately 40% of patients die or relapse within 3 years from diagnosis after standard one-size-fits-all immunochemotherapy R-CHOP (rituximab, cyclophosphamide, doxorubicin, vincristine, prednisone) [1,2]. As an explanation, DLBCL is generally assumed to be a complex disease with significant genetic heterogeneity resulting in different biological behavior and drug-refractoriness. Many studies examined the molecular background to understand the various mechanisms of lymphomagenesis and therapy resistance in DLBCL. Recurrently mutated genes corresponding to multiple pathways have been discovered demonstrating the intricate molecular background of DLBCL [3,4,5]. Despite these insights, an in-depth understanding of this biological heterogeneity is still lacking.

Over the past decades, analysis of the molecular background of DLBCL has advanced through gene-expression profiling (GEP) studies allowing for the investigation of cell-of-origin (COO), MYC expression and tumor microenvironment (TME). This review focuses on the emerging role of GEP studies in elucidating the biological heterogeneity of DLBCL, thereby improving diagnostic classification, prognosis, and ultimately the development of targeted treatment. Finally, to facilitate subsequent molecular studies in DLBCL, we propose a knowledge-based biology-driven and ready-to-use targeted GEP consortium panel, named BLYM-777, including probes targeting 777 genes, covering many aspects of lymphoma B cells and the TME.

## 2. Technical Approaches of Gene-Expression Profiling

**Figure 1:**
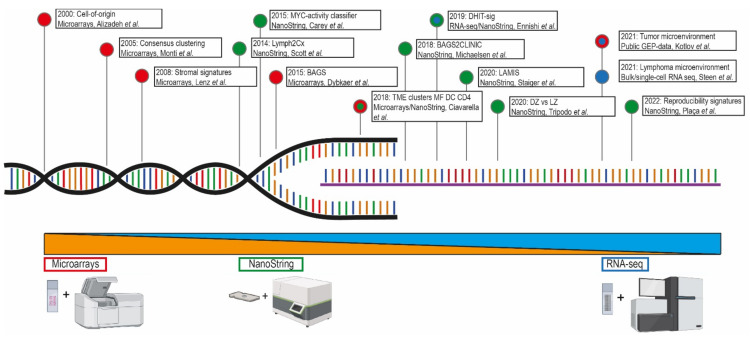
The meaningful arrival of GEP in DLBCL. This timeline presents the implementation of GEP strategies in DLBCL studies throughout the past two decades and marks the relevant findings with their corresponding techniques [7,9,10,11,12,13,14,15,17,18,19,20,21]. Within the lymphoma research field, technological advances shifted the approach from microarrays (red) to NanoString (green) and ultimately (single cell) RNA sequencing (blue).

Several GEP methodologies have been applied in DLBCL studies, as summarized in Table 1 and Figure 1. The most conventional technique is reverse-transcription quantitative polymerase chain reaction (RT-qPCR), in which mRNA is converted into complementary (c)DNA using reverse transcriptase and this cDNA is subsequently amplified using dyes or specific probes for quantification of the PCR product after each amplification cycle. This used to be a monogenic, labor-intensive method that was unable to screen multiple high-throughput transcripts, but major improvements in throughput have been made over the years allowing simultaneous amplification of multiple genes in parallel [6].

**Table 1:** Literature overview of relevant DLBCL studies with their respective GEP methods, number of included cases and genes, cluster targets and clinical relevance. COO = cell-of-origin, TME = tumor microenvironment, N.A. = not available, complete gene lists of these studies were not available.

First Author(s)	Year	GEP Method	No. of Cases	No. of Genes	No. of Genes in BLYM-777	Clusters	Clinical Relevance
Alizadeh, Elsen, et al. [7]	2000	Microarrays	47	2984	N.A.	COO	COO classified DLBCL into GCB or ABC with prognostic impact, possible benefit from different treatment options
Rosenwald, et al. [8]	2002	Microarrays	240	100	N.A.	GEP subgroups	COO classification into GCB and non-GCB (ABC and type 3), molecular predictor of survival after treatment
Monti, Savage, et al. [9]	2005	Microarrays	176	2118	97	Consensus clustering	Three identified DLBCL clusters; oxidative phosphorylation, BCR/proliferation or host response, no relation with survival
Lenz, et al. [10]	2008	Microarrays	414	382	60	Stromal signatures	Consensus clustering identified two stromal signatures predictive for survival and one GCB cluster
Alizadeh, Gentles, et al. [6]	2011	RT-qPCR	787	2	2	LMO2 and TNFRSF9	Two survival-correlated biomarkers and associated with TME
Scott, et al. [11]	2014	NanoString	119	20	20	COO	Validation of COO classification into GCB or ABC, reflecting survival, possible benefit from different treatment options
Carey, et al. [12]	2015	NanoString	55	200	33	MYC high- and low-risk clusterss	Classification and stratification of MYC-driven, aggressive BCL
Dybkær, Bøgsted, et al. [13]	2015	Microarrays	1139	223	37	B-cell associated gene signature (BAGS)	Further discrimination of COO in centrocytes, centroblasts, plasmablasts, or memory B cells, with survival outcomes
Ciavarella, Vegliante, Fabbri, et al. [14]	2018	Publicly available GEP-data and NanoString	482	45	45	TME clusters	TME classification presenting high prevalence of myofibroblasts, dendritic cells, or CD4 T cells related to survival outcomes
Michaelsen, et al. [15]	2018	NanoString	1058	128	53	BAGS2Clinic(expanded BAGS)	Intensified BAGS classification in centrocytes, centroblasts, plasmablasts, or memory B cells, predictive for survival
Davies, et al. [16]	2019	Illumina HiSeq sequencing	1076	N.A.	N.A.	COO	Molecular characterization for prospective stratification, randomization and analysis of DLBCL subgroups
Ennishi, et al. [17]	2019	RNA-seq	157	104	43	DHITsig	Defined GEP signature high-grade B-cell lymphoma double or triple hit with *BCL2* translocation
Staiger, Altenbuchinger, Ziepert, et al. [18]	2020	NanoString	466	145	17	Lymphoma-associated macrophage interaction signature (LAMIS)	Signature indicating the presence of macrophages and associated with poor survival
Tripodo, Zanardi, Ianelli, Mazzara, et al. [19]	2020	NanoString	551	87	52	Spatial dark- versus light-zone microenvironment signature	Distinguishing COO GCB subtype into dark or light zone with prognostic significance
Kotlov, et al. [20]	2021	Publicly available GEP-data	4580	203	144	Functional gene signatures and TME clusters	Four TME specific categories associated with survival and with opportunities for novel targeted treatment
Steen, et al. [21]	2021	Bulk/single-cell RNA sequencing	1584	20380	192	Cell states and ecotypes of the TME	Discrimination into cell types and cell states within the TME, correlated with survival, and facilitating development of new targeted treatment strategies

Over two decades ago, the application of a new gene expression profiling technique resulted in a hallmark study of DLBCL [7]. This microarray-based technology allows the simultaneous assessment of thousands of gene-transcripts. These microarrays contain probes that are complementary to fluorescently labelled cDNA produced by reverse transcription of mRNA from the genes of interest. After hybridization, digital cameras measure fluorescence intensity and translate this to gene-transcript counts. This method requires mRNA input from preferentially fresh frozen material over formalin-fixed paraffin-embedded (FFPE) samples. However, in routine diagnostics, the material is generally preserved in FFPE rather than fresh frozen due to practical considerations which impeded a broad implementation of studies using microarray-based technologies. Nowadays special FFPE kits (f.e., Agilent and Illumina) are available that allow the routine analysis of RNA isolated from FFPE.

The NanoString nCounter system (Seattle, WA 98109, USA) is an alternative hybridization-based gene expression profiling method. This technique detects and counts several hundreds of mRNA transcripts by using probe specific molecular “barcodes” combined with fluorescent-microscopic imaging. This system is efficient for targeted GEP strategies with (partially) degraded RNA samples (i.e., FFPE). After entering the lymphoma research field in 2014, the NanoString nCounter system has been widely used in studies to identify lymphoma subtypes.

With the advent of next-generation sequencing (NGS) techniques, RNA sequencing (RNA-seq) was developed as an alternative approach for GEP, enabling the analysis of entire transcriptomes. Besides the generation of gene-expression profiles, RNA-seq enables the analysis of gene fusions, mutations, single nucleotide polymorphisms, or even copy number alterations. For the generation of sequencing libraries, RNA is reverse transcribed to cDNA, and subsequently fragmented. Like microarray-based GEP assays, methodologies for RNA-seq using RNA isolated from FFPE has been developed and allows generation of reliable gene-expression profiles also from poor quality RNA. A derivative of RNA-seq is single-cell RNA-seq (scRNA-seq) for examination of the transcriptome of each individual nucleus as opposed to (tumor) bulk analysis. The main drawback of scRNA-seq is that massive data is produced that needs extensive bioinformatic procedures for appropriate analysis.

More comprehensive overviews of (dis)advantages of currently available technologies have been reviewed extensively by Narrandes et al. and Jiang et al. [22,23]. All of the above-described techniques, RT-qPCR, microarrays, NanoString, and (sc)RNA-seq, have been applied in DLBCL studies and are discussed below for their relevance to DLBCL pathogenesis.

## 3. The Arrival of Gene-Expression Profiling

**Figure 2:**
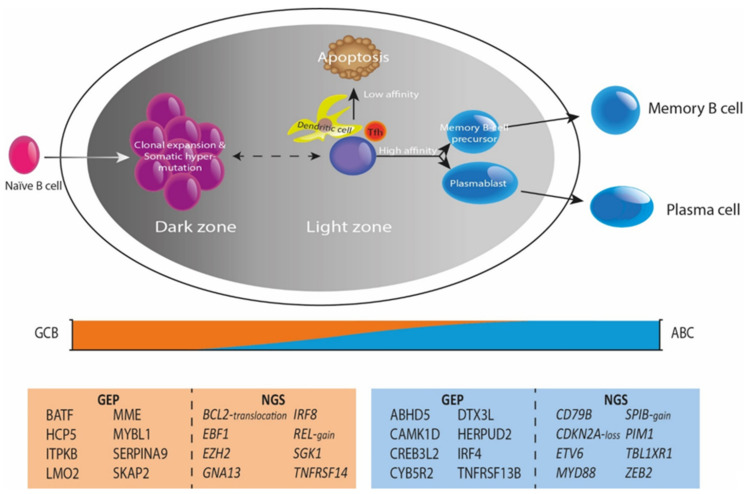
Genetic perspectives of B-cell lymphomagenesis. Under normal physiological circumstances, the germinal center is crucial for B-cell development and maturation, defining different cellular subtypes and states throughout this continuing process. DLBCL lymphomagenesis shows a GCB subtype in the earlier stages of development and an ABC subtype in later stages, representing COO classification. The COO classification is substantiated by distinct characteristic GEP and mutational profiles between GCB and ABC. This insight shows the importance of combining DNA NGS and GEP in a more multidimensional approach that improves classification and prognostication of DLBCL.

As presented in Table 1 and Figure 1, several relevant DLBCL studies reported on the use of gene-expression assays with different platforms. As a cornerstone, Alizadeh et al. [7] were the first in 2000 to demonstrate a large diversity between DLBCL cases in a microarray-based gene-expression study. This study defined two molecularly distinct DLBCL subtypes with either germinal center B-cell (GCB) or activated B-cell like (ABC) phenotypes, as shown in Figure 2. Tumors classified as GCB showed a significantly superior overall survival (OS) compared to ABC DLBCL cases. Accordingly, Rosenwald et al. [8] independently reported similar results with an additional third discriminating DLBCL subtype (designated as type 3), which has a similar survival rate as ABC DLBCL, and grouped together are generally referred to as non-GCB subtypes. These results were at the basis of identifying the COO to better understand lymphomagenesis. Several other studies aimed to optimize the GCB/non-GCB COO classification, explore other potential signatures for DLBCL, and validate previous findings [6,24,25,26,27].

The advent of the NanoString nCounter platform optimized gene-expression analysis of FFPE samples. Scott et al. [11] were the first to generate a COO classification using the NanoString technology in 2014. This approach utilized a targeted panel (Lymph2Cx), including 20 genes, and presented high intra-institutional concordance and overlap with microarray-based GEP. Subsequently, Dybkær et al. [13] aimed to further subdivide the classified COO subtypes of GCB and ABC into centrocytes, centroblasts, memory B-cells and plasmablasts. This initially led to the design of a microarray-based assay called B-cell associated gene signature (BAGS) including 223 genes, demonstrating a significantly different progression-free survival (PFS) and OS between the four cellular subtypes. Subsequently, in 2018, Michaelsen et al. [15] modified the BAGS assay to a new BAGS2CLINIC panel for the NanoString platform, including 128 genes, enabling fast and easy-to-use GEP with high overlap with the original BAGS classifier. Compared to the Lymph2Cx panel, the BAGS2CLINIC panel is more comprehensive and provides a more detailed stratification. Survival analyses using the COO assignment by BAGS2CLINIC indicated an inferior PFS and OS for the memory B-cell subtype compared to the plasmablast subtype, both originally classified as ABC subtypes. Although the centroblast and centrocyte subtypes were both classified as GCB subtypes, an inferior PFS was identified for the centroblast subtype, with no difference in OS.

In 2020, Tripodo et al. [19] generated a spatial signature including 87 genes that discriminates between the dark and light zone of the germinal center, with similarities to COO and BAGS(2CLINIC) classifications. The subtypes identified by this panel showed prognostic significance, as a light-zone-like phenotype was associated with superior OS compared to a dark-zone-like phenotype.

With the advancing insight into COO, interest and understanding of the TME have increased. In 2005 Monti et al. [9] analyzed transcriptional signatures in DLBCL and reported a so-called “consensus clustering” classification. This study implemented microarrays and sequential consensus cluster analysis to assess the stability of clusters in gene-expression data after different clustering methods. Three distinct DLBCL clusters were identified, two of which contained predominantly B-cell expression profiles characterized by oxidative phosphorylation and B-cell receptor/proliferation. In contrast, the third cluster was enriched for T-cell-mediated immune response and classical complement pathway and as such reflected the interaction of the microenvironment with the tumor. In contrast to COO, no correlation was found between the consensus clusters and survival [9].

In 2008, Lenz et al. [10,28] identified a GCB cluster and two stromal signatures, characterized by their TME association. The first stromal signature (stromal-1) reflected the extracellular matrix and histiocyte infiltration and was associated with a favorable PFS and OS in comparison to the second stromal signature (stromal-2) which represented tumor angiogenesis. Over the following years, other independent studies investigated these stromal signatures and other biological markers for their relevance to survival, reporting similar findings [21,26,29]. The identification of these stromal signatures emphasized the importance of studying the TME to improve the biological understanding of DLBCL [10].

Carey et al. [12] performed targeted GEP and identified a molecular classifier of MYC activity including 80 genes that stratified DLBCL patients into high- (MYC score > 0.5) and low-risk (MYC score < 0.5) groups. Patients with low MYC scores showed significantly better OS. This classification was further optimized by Ennishi et al. [17] who generated a double-hit gene-expression signature (DHITsig) including 104 genes. DHITsig positivity was determined by overexpression of genes of high-grade B-cell lymphoma double hit or triple hit with *BCL2* translocations. DHITsig-positive cases showed strong cell-autonomous survival and proliferation signals and reduced dependence on the TME. Using this DHITsig, approximately twice as many tumors were classified as high-grade B-cell lymphoma than with conventional fluorescence in situ hybridization (FISH). PFS and OS were significantly worse in DHITsig-positive patients in comparison to DHITsig negative patients. Plaça et al. [30] have successfully reproduced the MYC classifier of Carey et al. [12] and the consensus clustering of Monti et al. [9] in 175 samples of the HOVON-84 trial on a panel of 117 genes using the NanoString platform. These GEP signatures can facilitate the search for optimization of treatment algorithms, for example, which patients would benefit from the addition of lenalidomide to standard R-CHOP treatment (as described by Chamuleau et al. [31]) or to common intensive chemotherapy regimens.

In summary, along with technological advancements over time, several GEP signatures of lymphoma cells have been identified which have significantly augmented the biological knowledge of DLBCL, distinguishing several COO and TME-based molecular subtypes with prognostic relevance.

## 4. Integrating Gene-Expression Profiling and Mutational Profiles

DLBCL belongs to the spectrum of cancers with high mutational burden, reporting 7.8 driver mutations per case and a mean number of 23.5 mutations in ABC and 31 in GCB DLBCL patients, respectively [3,4,32,33,34,35]. Using (targeted) DNA (t)NGS technologies, from now on referred to as NGS, multiple studies identified the involvement of various intracellular signaling cascades (f.e., apoptosis, DNA damage response, JAK/STAT, MAPK, NF-κB, NOTCH, PI3K) in DLBCL lymphomagenesis. Karube et al. [36] defined the relevance of genomic alterations in genes involved in the NOTCH pathway in DLBCL suggesting that analysis of aberrations in defined pathways may be more instructive than independent genes alone. Recently, several large NGS studies have shown that various molecular subgroups informative for prognosis can be distinguished in DLBCL [4,5,37,38,39,40]. In 2018, Chapuy et al. [4] identified five robust molecular DLBCL subgroups, C1-C5. Similarly, Schmitz et al. [5] identified four distinct subtypes, MCD (co-occurrence of *MYD88^L265P^* and *CD79B* mutations), BN2 (*BCL6* fusions or *NOTCH2* mutations), N1 (*NOTCH1* mutations), and EZB (*EZH2* mutations or *BCL2* translocations). Wright et al. [37] revealed six genetic subtypes, including the four subtypes that Schmitz et al. already reported, supplemented by the A53 (*TP53* mutations) and ST2 (*SGK1* and *TET2* mutations) subtypes, known as the LymphGen profiles. Similarly, Lacy et al. [38] identified five molecular subgroups, *MYD88, BCL2, SOCS1/SGK1, TET2/SGK1, NOTCH2* and an unclassified group. From these large sequencing studies at least five distinct molecular subgroups have been defined, partially representing COO subtypes (Figure 2); *MYD88/CD79B* (NF-κB pathway), *TP53*, *BCL2/NOTCH2*, *SOCS1/SGK1* (JAK/STAT pathway) and *MYC*.

While the pathogenicity of most aberrations on lymphomagenesis is well understood, for a deeper understanding of DLBCL biology it remains of major importance to complement these molecular profiles with gene-expression profiles. An elegant example is a key study by Steen et al. [21], that identified different B-cell states by GEP and integrated this with genomic data, by comparing the B-cell states to the results of the genomic LymphGen profiles and the C1-C5 subtypes [4,37]. This comparison resulted in a partial overlap between these two different subtyping methods and the identified B-cell states, but also revealed significant differences between these mutational and gene-expression classifications. These differences showed that tumors within similar mutational profiles differed in their transcriptional profile and depend on different effects on downstream pathways.

This concept was underscored by Shouval et al. [41], who identified two complementary mechanisms in *TP53*-mutated DLBCL using transcriptomic profiling. The first mechanism was the downregulation of IFN signaling and the second was characterized by a reduced tumor infiltration of CD8-positive T cells. Both mechanisms contributed to treatment resistance and thereby to inferior survival. This approach demonstrated that *TP53*-mutated DLBCL could be further subdivided by transcriptomic profiling improving the understanding of clinical behavior or treatment responses of these tumors. 

As summarized above, Figure 2 shows that GEP and NGS data complement each other and provide clear added value in understanding the complicated molecular subtypes of DLBCL, for example, for further subtyping of COO classes. This strategy has shifted the field of research towards a more multidimensional approach connecting NGS and GEP data from individual DLBCL cases across the entire study cohort.

## 5. The Tumor Microenvironment as Defined by Gene-Expression Profiling

**Figure 3:**
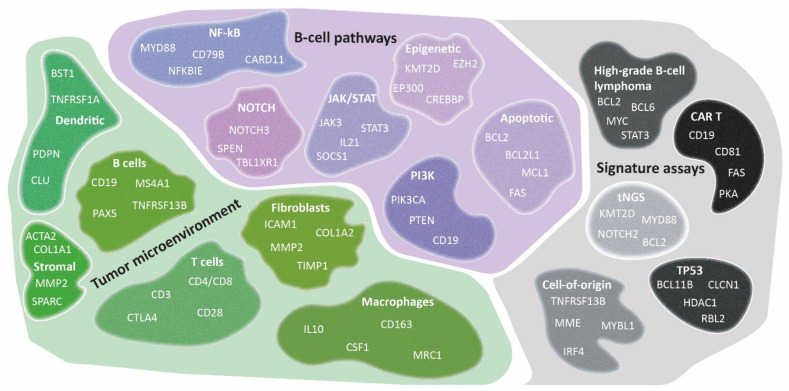
Diversity of TME signatures in DLBCL. Several GEP signatures of lymphoma cells have been identified that have significantly augmented the biological knowledge of DLBCL. As presented, these signatures could be subdivided into three categories: tumor microenvironment, B-cell pathways, and signature assays. A relevant gene selection of potential pathways related to B-cell lymphomagenesis (purple), cell types within the TME (green), and other specific signature assays (grey) are depicted. GEP studies have demonstrated its added value in characterizing the DLBCL microenvironment and the discovery of early principles of their intriguing mechanisms. However biological issues remain, and further research is needed to determine the true clinical benefit.

The development of GEP technologies has offered the possibility to study the TME more extensively. To address various biological and clinical questions, GEP approaches have primarily focused on the role of fibroblasts, macrophages, or T cells in DLBCL lymphomagenesis. Targeted panels with probe sets covering genes encompassing discriminatory aspects of fibroblasts, macrophages, T cells, or other cells, and their activation and differentiation states have been utilized. With this TME-directed GEP strategy, several TME signatures have been defined in DLBCL, as presented in Figure 3.

Analysis of GEP data of 414 untreated DLBCL samples identified three distinct gene-expression signatures; GCB, stromal-1, and stromal-2 [10]. The stromal-1 signature was associated with favorable survival. A computational CIBERSORT method incorporating 17 immune and stromal cytotypes into a 1028-gene matrix was applied to the previously produced data [10,14,42]. This analysis revealed that a high prevalence of myofibroblasts, dendritic cells, or CD4-positive T cells was associated with superior PFS and OS as compared to an abundance of activated natural killer cells and plasma cells. Subsequently, a 45-gene set developed for NanoString-based profiling demonstrated a favorable survival of DLBCL with a high prevalence of these similar cell types [14].

Furthermore, a lymphoma-associated macrophage interaction signature (called LAMIS, including 145 genes) was developed that specifically targets macrophages with the M2 phenotype that are immunosuppressive and promote tumor progression [18]. High expression of the LAMIS-signature indicated poor PFS and OS, independent of COO subtype and International Prognostic Index (IPI) score [14,18]. Accordingly, Marcelis et al. [43] characterized the TME of primary central nervous system lymphoma and reported that an increased M1-like/M2-like macrophage ratio was associated with superior OS. Keane et al. [44] quantified the TME independent of the revised IPI and COO by evaluating the ratios of immune effectors with potential implications for the selection of patients in clinical trials.

Two novel corner stone studies investigated the TME through GEP and correlated these data with clinical and NGS data. Initially, in 2021, Kotlov et al. [20] performed clustering analysis on a large dataset retrieved from several publicly available datasets (*n* = 25, 4580 DLBCL cases). Based on functional gene signatures, four different cellular subtypes of the lymphoma microenvironment were clustered: germinal center-like, mesenchymal, inflammatory, and depleted. The first cluster was characterized by germinal center features, the second cluster showed a high abundance of stromal cells and extracellular matrix pathways, the third cluster was associated with inflammatory pathways, while the depleted cluster lacked markers of these three defined GEP signatures. These lymphoma microenvironment clusters showed an impact on PFS and OS regardless of COO or genetic subtype, underlining its independent contribution to lymphomagenesis and clinical presentations. These clusters also demonstrated large similarities with previously discussed COO, consensus clustering, stromal signatures, and genetically defined entities (LymphGen profiles and C1-C5 subtypes) [4,5,20,37].

Simultaneously, as another landmark, Steen et al. reported on their so-called “Ecotyper” -algorithm that was generated for either solid tumors or lymphomas (mainly DLBCL). The landscapes of (tumor) cell states and lymphoma ecosystems were examined by means of bulk or single-cell RNA-sequencing. In this study, the B-cell states represented the previously discussed COO including GCB and ABC subtypes, as well as the subdivision into centrocytes, centroblasts, memory B-cells, and plasmablasts established by the BAGS2CLINIC. Accordingly, these B-cell states were associated with survival, corresponding to the COO or BAGS2CLINIC classification, and molecular classification with the LymphGen and C1-C5 clusters [4,37]. Furthermore, other cell-type states were identified along with a total of 9 lymphoma ecotypes that congregated multiple cell-type states and were equally associated with survival outcomes [21]. Thus, Kotlov et al. [20] and Steen et al. [21] individually identified distinct microenvironment subtypes associated with molecular profiles and survival outcomes.

As summarized in Figure 3, complementing GEP studies focusing on tumor cell subtyping and the lymphoma TME contributed to the insight that several cellular subtypes within DLBCL phenotypes were related to survival. Conclusively, an association is shown between the presence of high numbers of M2-type macrophages, natural killer cells, plasma cells, or increased angiogenesis with inferior survival. In contrast, abundant infiltration of myofibroblasts, dendritic cells, CD4-positive T cells, CD14-positive monocytes, extracellular matrix deposition and histiocytes demonstrated superior survival. However, results should be interpreted cautiously because validation studies are lacking.

The prognostic impact of the TME signatures does not answer the question of whether these microenvironmental features indicate an underlying interaction of infiltrating cells with tumor cells that promote tumor growth or, in contrast, represent an ultimate consequence of cellular damage and is thus initiated by the tumor itself. GEP studies have demonstrated their value in mapping the DLBCL microenvironment. Nevertheless, unresolved biological issues on the interaction and activation status of diverse cell types in the TME remain and further research is needed to determine the true clinical effect of the interaction between lymphoma cells and the TME.

## 6. Clinical Impact and Future Perspectives of Gene-Expression Profiling Studies

**Figure 4:**
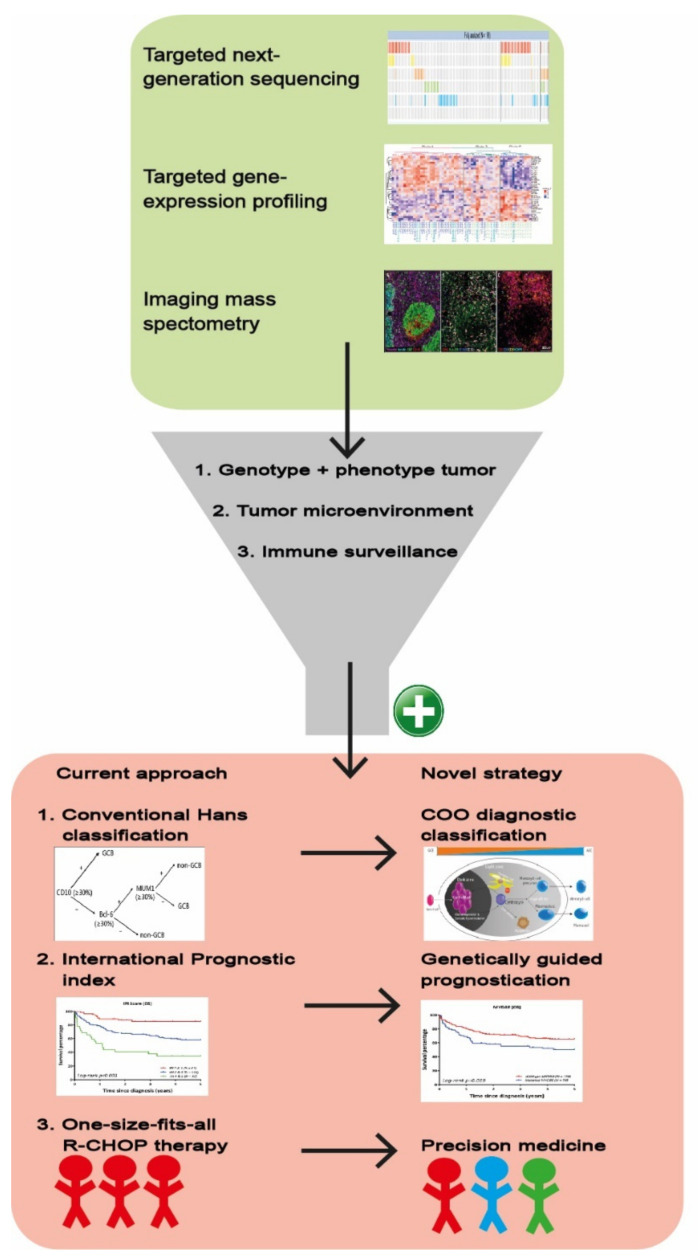
Schematic overview of a multilayered research strategy. Combining targeted NGS, targeted GEP and imaging mass spectrometry allows for inclusive analysis of genotype, phenotype, TME and immune surveillance of the DLBCL. This methodology substantiates the conversion from the current approach towards a novel strategy including (1) Hans classification to COO diagnostic classification, (2) a general clinical prognostic score (International Prognostic Index) towards a biology-guided prognostication and ultimately (3) facilitating development from a one-size-fits-all R-CHOP treatment towards more precision medicine.

As depicted in Figure 4, GEP analysis has the potential to elucidate the phenotype of the tumor, the composition of the TME, and the presence of immune surveillance mechanisms. Consequently, GEP studies have refined current DLBCL categorization towards a more biologically driven classification with different COO subtypes. Besides a more general consensus clustering, the GEP COO classification into ABC and GCB subtypes has changed the view of DLBCL’s biological behavior and is steadily regaining its place in diagnostic procedures above the surrogate Hans algorithm for COO based on immunohistochemical staining [27]. In addition, GEP COO classification is used to allocate patients with particular DLBCL subtypes to novel targeted clinical trials although a true clinical benefit has not yet been established [16,45,46,47].

COO classification has been further developed into dark-zone-like and light-zone-like phenotypes or even more specific cell types, such as centrocytes, centroblasts, memory B-cells and plasmablasts, all harboring a prognostic impact. Other independently identified GEP clustering studies in DLBCL demonstrated predictive significance, such as stromal, immune-related, LAMIS, lymphoma microenvironment, “Ecotyper”, and other cellular-specific signatures (Table 1). However, despite the significant progress made in the refinement of these biological and predictive classifications, validation studies for these prognostic signatures are currently lacking and hinder direct implementation in routine clinical settings to optimize patient management and counseling. Besides that, GEP techniques are time consuming, many pathology departments are not equipped with these technologies, lack biostatistical tools needed for examining these signatures, and costs are currently not covered by most health insurances. Although optimizing the specificity of DLBCL classification is crucial to improving patient care, the challenge is to arrive at a consortium-oriented panel for discriminatory subtyping that is clinically relevant, easily accessible, has a short turnaround time, and is affordable for routine use.

GEP results can also be used to initiate a new era of therapeutic trials. Given the intermediate response to R-CHOP in DLBCL, better subtype classification of mainly high-risk DLBCL subgroups such as high-grade B-cell lymphoma could improve the effectiveness of targeted therapeutic strategies. Targeted therapies that complement or replace standard treatment have been investigated. For example, Davies et al. (2019) [16] studied the effect of adding bortezomib, a proteasome inhibitor, to R-CHOP treatment and reported no significant improvement in survival. Data retrieved from this trial, have been re-examined, identifying a DLBCL subgroup characterized by the prevalence of a distinct CD8 T-cell state that benefited from the addition of bortezomib to R-CHOP [21]. Therefore, further patient classification depending on COO status or particular signatures could improve the efficacy of bortezomib by more efficient upfront patient selection [48,49].

Kuo et al. [45] revealed that ibrutinib was less effective in ABC subtype DLBCL patients with high *BCL2* expression. Wilson et al. [50] investigated this in more detail and reported that for patients of <60 years the event-free survival after treatment with ibrutinib and R-CHOP was 100% in the MCD (co-occurrence of *MYD88^L265P^* and *CD79B* mutations) and N1 (*NOTCH1* mutations) subtype. Hartert et al. [51] described the favorable effect of adding lenalidomide to R-CHOP on event-free survival in patients with mutations in *PIM1, SPEN* or *MYD88* or expression signatures including NF-κB, IRF4 and JAK-STAT. The addition of venetoclax to R-CHOP treatment reported worse outcomes than expected in patients overexpressing *BCL2*, underlining the necessity of analyzing involved pathways [52,53]. These examples show that intensifying molecular analysis is needed for the optimization of personalized treatment [54]. Consequently, this paves the way to re-evaluate previous clinical trials adopting targeted therapies, potentially providing new insights into the current conclusions.

Another important application of GEP results is in the management of resistance and efficacy of CAR T-cells and bispecific antibodies in DLBCL patients. These treatments have shown remarkable efficacy in chemorefractory DLBCL patients; however, in a significant proportion of patients, these therapies are still not effective [55,56,57,58,59]. Critical analysis of the TME and its influence on the effectivity of CAR T-cell and bispecific antibody therapy in DLBCL is lacking. GEP studies in DLBCL patients focusing on the TME will facilitate further evaluation of the disparate response to these novel treatments.

Kahle et al. [60] published a review on the contribution of molecular imaging to the understanding of the biology of lymphoma. Adding immunohistochemistry, proteomics, or imaging mass spectrometry to NGS and GEP data enables a multi-dimensional analysis of tumors related to the TME. For example, de Miranda et al. [61,62,63] performed imaging mass cytometry on tonsil and colorectal cancer tissues, thereby enhancing the understanding of the heterogeneous and intricate tumor-specific immune landscape of TME. Such molecular imaging analysis can complement current molecular evaluations in DLBCL to a more three-dimensional analysis, facilitating the identification of new mechanistic concepts. In addition, analysis of paired samples before and after an intervention will deepen the analysis of the TME, subclones and (acquired) therapeutic resistance [64]. The application of machine learning tools such as artificial intelligence will further enhance the utility of multi-omics data to better define distinct molecular and prognostic DLBCL subtypes [65].

In summary, to improve the understanding of the intricate molecular biology of DLBCL and the interaction with its TME, future studies should adopt a multi-dimensional strategy including immunohistochemistry, NGS, GEP and proteomics. Figure 4 shows such a multilayered approach and emphasizes that in addition to appropriate equipment, it also requires a diverse and well-trained team of molecular biologists, pathologists, hematologists, bioinformatics, and biostatisticians. Relevant findings of this multilayered analysis will ultimately be translated into manageable diagnostics that can be implemented by multiple medical centers.

## 7. Anatomical Localization and Age Matter

**Figure 5:**
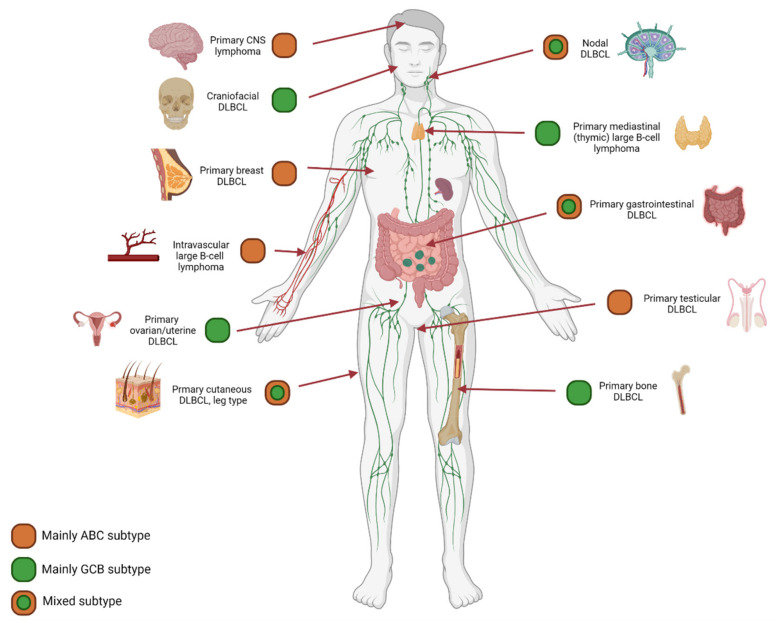
COO subtype: Anatomical localization matters. The results of diverse studies using GEP or Hans classification for COO determination demonstrated an evident association between anatomical preferred localization and COO subtype. For example, primary central nervous system lymphoma, primary testicular lymphoma, and intravascular large B-cell lymphoma harbor predominantly an ABC subtype. In contrast, we recently demonstrated a GCB subtype for primary bone DLBCL, that could be specified even further to unique cellular phenotypes [67]. This concept calls for additional investigation of well-annotated homogeneous cohorts of preferred localization DLBCL, including in-depth molecular studies.

Together with other NGS studies, we demonstrated unique mutational profiles for DLBCL with a preferred localization, f.e., in primary central nervous system B-cell lymphoma, primary testicular lymphomas, intravascular large B-cell lymphoma, primary bone DLBCL, and primary cutaneous DLBCL leg-type [66,67,68,69,70,71,72,73]. These preferred anatomical localized DLBCLs were significantly associated with specific COO subtypes variably determined by GEP and Hans classification (Figure 5). Primary central nervous system B-cell lymphoma, primary breast DLBCL, intravascular large B-cell lymphoma, and primary testicular large B-cell lymphoma are mainly classified as ABC type lymphomas. In contrast, craniofacial, primary mediastinal (thymic) large B-cell lymphoma, primary ovarian DLBCL and primary bone DLBCL are mainly classified as GCB [67,74,75]. In addition, the Lymph3Cx GEP panel was developed as an update of the Lymph2Cx, which has distinguished primary mediastinal (thymic) large B-cell lymphoma from other DLBCL subtypes [75]. By applying a targeted NanoString panel we recently demonstrated that primary bone DLBCL mainly constitutes a centrocyte-like GCB-profile, while non-osseous DLBCL with a GCB subtype principally has a centroblast-like phenotype [67]. This conceptualizes that anatomical DLBCL localization is relevant for specific COO subtypes and even more for unique cellular phenotypes. In short, for COO subtypes, localization matters.

Another correlation was seen between age and COO subtype, as in the elderly an ABC subtype was predominant, indicating that COO follows the physiology of senescence and alteration of the T-cell repertoire [76,77]. Altogether, these concepts have further broadened the molecular view of DLBCL, as these techniques allow COO to even be considered down to the individual cell level. As we have mentioned earlier, these results confirm the additional value of exploring well-annotated homogeneous cohorts and appeal to the need for in-depth molecular studies of DLBCL with preferred localization [78].

## 8. A Proposal for a Consortium Gene-Expression Profiling Panel: BLYM-777

**Figure 6:**
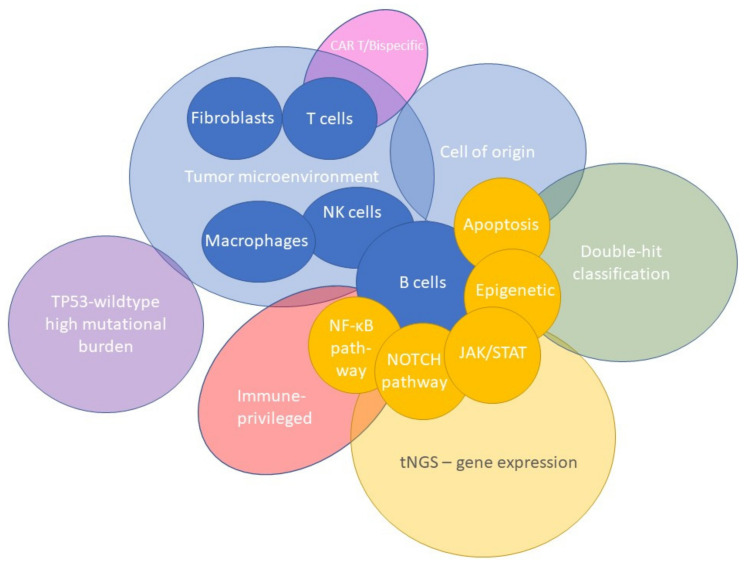
A proposal for a targeted BLYM-777 consortium panel. Based on 45 studies, we propose a knowledge-based, biology-driven targeted (t)GEP consortium panel, called BLYM-777. This BLYM-777 panel primarily focuses on DLBCL and covers 777 B-cell lymphoma relevant genes, including their involved pathways (f.e., NF-κB, NOTCH, PI3K). Accordingly, genes were included for COO identification, TME-focused signatures, ecotypes, DHITsig, differentially expressed genes found in wildtype-*TP53* DLBCL, and genes relevant for resistance to CAR T-cell or bispecific antibody therapy. Moreover, 95 genes important for current molecular classification based on NGS results have been included.

From a biological point of view, it is important to apply GEP analysis as broadly as possible. In practice, this is often not feasible, for instance if only limited amounts of archived FFPE material are available. For this reason, a targeted GEP approach is frequently used because it is clinically applicable and allows the analysis of a limited set of genes of interest. Based on reviewing more than 45 studies and considering the maximum number of 800 genes that can be analyzed using NanoString technology, we generated a targeted knowledge-based biology-driven (t)GEP consortium panel, called BLYM-777 (Figure 3 and Figure 6 and Appendix A). This BLYM-777 panel includes 777 genes involved in the NF-κB (f.e. *MYD88*, *CD79B* and *CARD11*), JAK/STAT (f.e., *SOCS1, JAK1* and *STAT1*), MAPK (f.e., *BCL2* and *MEK2)*, NOTCH (f.e., *NOTCH3* and *TBL1XR1)*, PI3K (f.e., *PTEN* and *PI3K)* pathways that are known to be important in lymphomagenesis of DLBCL. In addition, the BLYM-777 includes genes relevant for COO identification, such as the original COO-classification NanoString tool Lymph2Cx (*n* = 20 genes) (f.e., *IRF4, ITPKB, MME* and *MYBL1)*, the BAGS2CLINIC (*n* = 53) (f.e., *STAT3* and *IL16)*, and dark-/light-zone signature (*n* = 52) (f.e., *B2M, CTLA4, KI67* and *AICDA*), all of which have individually been shown to facilitate DLBCL subtype classification [11,15,19]. As the interest in the TME increases in DLBCL, BLYM-777 additionally includes genes related to TME-focused signatures, such as the consensus clustering classification (*n* = 86) (f.e., *CD37, TNFRSF1A* and *PDL1)*, LAMIS signature (*n* = 63) (f.e., *CCND2* and *CXCR4)*, a 45-gene TME assay (Ciavarella et al. *n* = 45) (f.e., *COL1A1* and *MMP2)*, lymphoma microenvironments (*n* = 155) and ecotypes (*n* = 314) [9,14,18,20,21]. This BLYM-777 design also covers other signatures relevant for DLBCL, such as the DHITsig (*n* = 35) (f.e., *ETV6* and *RGCC)* since DHIT lymphomas show an inferior survival, genes relevant in MYC driven B-cell lymphomas (*n* = 80) (f.e., *RFC3* and *TRAP1)*, genes upregulated in wildtype-*TP53* DLBCL with high mutational burden (*n* = 37) (f.e., *HDAC1* and *BBC3)*, and genes relevant for the identification of resistance to CAR T-cell or bispecific antibody treatment (*n* = 35) (f.e., *CD58* and *FOXP1)* [12,17,55,56,57,79,80,81,82,83,84,85,86,87,88]. To evaluate the influence of mutations on gene expression, 95 genes important for current molecular classification based on NGS results have also been included [67]. Supplementary Table S1: BLYM-777 included genes per author, lists all genes belonging to this BLYM-777 tGEP consortium panel. To approach multiple biologically and clinically relevant questions on the lymphomagenesis of DLBCL, such as the relevance of certain mutations, the interaction of malignant cells with different components of the TME, immune surveillance and effectivity of new treatment strategies, the proposed BLYM-777 panel can be deployed in combination with other molecular characterization techniques. In addition, BLYM-777 is ready to use with NanoString technology and benefits from low-threshold accessibility and good performance using RNA isolated from FFPE material. However, analysis of the proposed panel of 777 genes is also possible by selective bioinformatic analysis of whole transcriptome data or targeted expression data generated by other platforms. The advantages and disadvantages of different gene-expression detection technologies have been extensively described by Narrandes et al. and Jiang et al. [22,23] and are beyond the scope of this clinical translational review.

In summary, the BLYM-777 panel covers many aspects of B-cell lymphomagenesis, COO classification, therapeutic efficacy and TME-focused signatures and can facilitate subsequent molecular investigations. The authors of this publication are currently in discussion with NanoString with the goal to create this consortium gene-expression BLYM-777 panel capturing the biology mentioned in this work. Such a panel could bring value to the hematological field by providing a standardized tool to facilitate collaboration and shared learnings throughout the community. If you are interested in joining this consortium effort, please respond to the corresponding author for more information.

## 9. Conclusion

This review provides a comprehensive overview of current molecular insights into the biological background of DLBCL obtained by several GEP technologies. These methods utilized in DLBCL studies identified several GEP signatures including cell-of-origin discrimination in GCB and ABC subtypes and an in-depth analysis of the TME regarding the exact cell type and state. Combining GEP with other NGS and proteomic-based methodologies will facilitate a multi-layered analysis and a next step forward in understanding biological principles and elucidating the genetic heterogeneity of DLBCL. The proposed novel knowledge-based biology-driven consortium tGEP panel, named BLYM-777, encompasses many aspects of B-cell lymphomagenesis, TME and immune surveillance and is thereby expected to gain new molecular concepts of DLBCL lymphomagenesis. Applying BLYM-777 in such a multilayered methodology potentially enhances diagnostic classification of DLBCL subtypes, prognostication, and ultimately the development of novel targeted therapeutic strategies improving patient survival.

## Supplementary Materials

The following supporting information can be downloaded at: https://www.mdpi.com/article/10.3390/cancers14081857/s1, Table S1: BLYM-777 included genes per author.

## Appendix A

**Table A1:** A proposal for a consortium gene-expression profiling panel: BLYM-777.

ACTA2	CKAP4	HIST1H2BC	MPST	SBK1
ACTB	CLCN1	HLA-A	MRC1	SCNN1D
ACTG1	CLCN2	HLA-B	MRC2	SCOTIN
ACTG2	CLU	HLA-C	MRPL15	SAMD13
ACTL7A	COL12A1	HLA-DMA	MRPL3	SELPLG
ADA	COL1A1	HLA-DMB	MRPL33	SEMA7A
ADHFE1	COL1A2	HLA-DPA1	MRPS34	SEP15
AEBP1	COL3A1	HLA-DPB1	MS4A1	SERPINA1
AEN	COL4A1	HLA-DQA1	MSH3	SERPINA9
AFMID	COL4A2	HLA-DQB1	MSR1	SERPING1
AGER	COL5A2	HLA-DRA	MUC16	SGK1
AGR2	COL6A3	HLA-DRB1	MXRA5	SGPP2
AHCY	COMMD8	HLA-E	MYBBP1A	SH2D1A
AHR	COX7A2L	HMG20A	MYBL1	SH2D1B
AICDA	CPD	HNRNPLL	MYBL2	SH2D3C
AKAP1	CPNE3	HPDL	MYC	SH3PXD2A
AKAP5	CPT1A	HRK	MYD88	SHARPIN
AKR1D1	CREB1	HS3ST3A1	NBR1	SHISA8
ALCAM	CREB3L2	HSPBL2	NCAM1	SIGLEC9
ALDH3B1	CREBBP	HTR1A	NCR1	SIK1
ALOX5	CSF1	ICAM1	NCR3	SIPA1L3
AMT	CSF1R	ICOS	NDUFB1	SKAP2
ANAPC16	CTHRC1	IDH1	NEMF	SLAMF1
ANGPT1	CTLA4	IDO1	NFAM1	SLAMF8
ANGPT2	CTNNA1	IFITM1	NFATC2	SLC12A8
ANO9	CTNNB1	IFNA16	NFKB1	SLC16A9
ANTXR2	CTPS1	IFNAR1	NFKB2	SLC25A27
AP1B1	CTSB	IFNG	NFKBIE	SLC29A3
APLP2	CTSK	IGHM	NKG7	SLC2A3
APOL6	CTSZ	IGLL3	NME1	SLC41A1
APRIL	CX3CL1	IGLL5	NOD2	SLFN5
ARG1	CX3CR1	IGSF10	NOLC1	SMAD1
ARHGAP17	CXCL10	IGSF6	NOTCH3	SMARCA5
ARID1B	CXCL11	IK	NPFF	SMIM14
ARSI	CXCL12	IKZF2	NPFFR2	SNHG19
ASB13	CXCL13	IKZF4	NR4A2	SOCS1
ASNSD1	CXCL5	IL10	NRF1	SOD1
ASPH	CXCL8	IL15	NRN1L	SP3
ATM	CXCL9	IL16	NSA2	SPARC
ATP5D	CXCR2	IL18BP	NSUN2	SPEN
ATRAID	CXCR3	IL1R1	NSUN5	SPI1
AURKA	CXCR4	IL2	NTRK1	SPIB
AURKB	CXCR5	IL21	OAZ1	SPP1
B2M	CYB5R2	IL21R	OPA1	SRM
BATF	DAB2	IL22	OR13A1	SSBP3
BATF3	DBI	IL2RA	OR4D5	STAM
BAX	DBP	IL2RB	OSBPL10	STAP1
BBC3	DDX11	IL4	OSMR	STAT1
BCAS4	DDX21	IL4I1	OTULIN	STAT2
BCL10	DDX6	IL6	OXTR	STAT3
BCL11B	DHRS2	IL6R	P2RY12	STAT6
BCL2	DHX33	IL6ST	P2RY14	STAU1
BCL2A1	DKK3	IL7R	PABPC3	STC2
BCL2L1	DNAJB12	INHBA	PAICS	SULF1
BCL2L12	DPP8	INPP5D	PALLD	SYBU
BCL6	DPYSL3	INSM2	PAPSS2	SYNE1
BCL7A	DTX1	IQCD	PARP1	TADA2B
BCLAF1	DTX3L	IRF1	PARP3	TAP1
BGLAP	DUSP2	IRF2BP2	PATL2	TAP2
BGN	DUSP4	IRF4	PAX5	TBL1XR1
BID	DUSP5	IRG1	PAX8-AS1	TBP
BIRC2	E2F1	IRS2	PCDH9	TBX21
BIRC3	EARS2	ISY1	PCLAF	TCIRG1
BLK	EBER1	ITGA6	PCNP	TCL1A
BRAF	EBER2	ITGB2	PCOLCE	TCP10
BSG	EBF1	ITGB8	PDCD1	TEDC2
BST1	EBI3	ITK	PDCD10	TEK
BTBD3	EBNA1BP2	ITM2A	PDCD1LG2	TESPA1
BTC	EEPD1	ITPKB	PDE5A	TET2
BTG1	EGFR	ITPR2	PDGFC	TGFBI
BTG2	EGR1	JAK1	PDGFRB	THBS2
BUB1	EGR3	JAK2	PDPN	THPO
C10orf128	ELL2	JAK3	PDXDC1	TIGIT
C14orf70	EMCN	JAKMIP1	PECAM1	TIM3
C16orf54	EOMES	JAML	PEG10	TIMP1
C17orf56	EP300	JCHAIN	PERP	TIMP2
C19orf24	EPHA4	KCNA4	PGF	TIMP3
C2	ERCC2	KCNH4	PHB2	TINAGL1
C3AR1	ERN1	KCNU1	PHF23	TJP1
C3orf22	ESCO2	KDR	PIK3CA	TLR8
C3orf37	ETFA	KI67	PILRA	TMEM119
CA9	ETS1	KIAA1128	PIM1	TMEM127
CABP2	ETV6	KIAA1462	PIM2	TMEM135
CACNA1I	EZH2	KIF14	PKA	TMEM140
CACNA2D2	EZR	KIR2DL4	PLCG2	TMEM175
CADM4	F8A3	KIT	PLCH2	TMEM202
CALR	FABP5	KLF2	PLD3	TMEM219
CAMK1D	FADD	KLHL14	PLK1	TMEM224
CAPS	FAM108C1	KLHL6	PLOD2	TMEM30A
CARD10	FAM117B	KLRC2	PMP22	TMEM47
CARD11	FAM13AOS	KLRF1	PMPCB	TMEM97
CARD14	FAM153A	KLRK1	PMS2P2	TMSB4X
CARD9	FAM216A	KMT2D	PMS2P9	TNF
CASP10	FAM26F	KRAS	POLD2	TNFAIP3
CASP8	FAS	KRT73	POLH	TNFRSF10B
CBLB	FASLG	LAG3	POLR1B	TNFRSF13B
CCDC154	FASN	LAMB1	POSTN	TNFRSF13C
CCDC50	FAT4	LAMP1	POTEC	TNFRSF14
CCDC6	FBL	LDHB	POU6F1	TNFRSF17
CCL20	FBLN2	LGALS7	PPAT	TNFRSF18
CCL4	FBLN7	LGALS9	PPP1R3B	TNFRSF1A
CCL5	FBXW7	LIMD1	PPRC1	TNFRSF1B
CCNB1	FCER1G	LINC01215	PRDM1	TNFRSF4
CCND1	FCGR1B	LMO2	PRDX5	TNFRSF9
CCND2	FCRL5	LOC100128071	PRKCH	TNFSF13B
CCND3	FDCSP	LOC100128682	PRKCQ	TNFSF8
CCNE1	FEM1C	LOC100131225	PRMT1	TOX
CCR6	FGD5	LOC100131354	PRNP	TP53
CCR8	FGFBP2	LOC100287094	PSAT1	TPO
CD11C	FIBP	LOC100287259	PSEN1	TPT1
CD160	FLJ37307	LOC100287308	PSIMCT.1	TRAC
CD163	FLJ37786	LOC100288639	PSMA2	TRAF1
CD19	FLT1	LOC100288728	PSMA5	TRAF2
CD2	FN1	LOC100289566	PSMA6	TRAP1
CD20	FNDC1	LOC196415	PSMB10	TRAT1
CD22	FNDC3B	LOC284889	PSMB9	TRBC1
CD226	FOXJ3	LOC391358	PSMD14	TRIAL-R1
CD24	FOXP1	LOC401433	PSMD3	TRIM21
CD244	FOXP3	LOC440311	PTEN	TRIM56
CD274	FSTL1	LOC729535	PTGES2	TRRAP
CD276	FYB	LRP12	PTPN11	TSKU
CD28	FYN	LRP1B	PTPN13	TSPAN9
CD300LF	GABRB1	LRP8	PTPRC	TTC8
CD37	GAMT	LSM1	PTTG1IP	UBA1
CD39	GATA2	LTB	QRSL1	UBASH3A
CD3D	GATA3	LTBR	R3HDM1	UBE2D2
CD3E	GATAD2B	LUM	RAB27A	UBXN4
CD3G	GBP1	LY6E	RAB29	UBXN7
CD4	GBP5	LY75	RAB33A	UCHL3
CD40	GDF2	LYAR	RAB3GAP2	UCK2
CD40LG	GEMIN4	MAF	RAB7A	VASP
CD44	GIT2	MAFB	RABEPK	VCAM1
CD47	GLRX	MAG	RAD54L	VEGFA
CD58	GNA13	MALT1	RAG2	VEGFB
CD6	GNAI2	MAML3	RANBP1	VEGFC
CD68	GNG12	MAP2	RASA1	VISTA
CD70	GNLY	MAP2K2	RASGRF1	VPS24
CD79A	GOT2	MAP4K1	RASL11A	VRK3
CD79B	GPNMB	MARCKSL1	RBL2	VTN
CD80	GPR124	MCL1	RBPJL	VWF
CD81	GPR137B	MCM2	REL	WAC
CD83	GPRIN3	MCM6	RELA	WASH2P
CD84	GRHPR	MDFIC	RELB	WASL
CD8A	GRIN3A	MDM2	RFC3	WDR3
CDC25A	GRN	MED23	RFFL	WDR55
CDCA7L	GSK3B	MEF2B	RGCC	XBP1
CDH23	GUK1	MEX3C	RNF130	XRCC3
CDH5	GZMB	MFAP2	RNF213	XRCC5
CDK2	GZMH	MFGE8	ROCK1	ZBTB4
CDK4	HDAC1	MIF	RPLP0	ZEB2
CDK5R1	HERPUD2	MIR155HG	RRS1	ZFAND4
CDKN2A	HIF1A	MME	RUBCNL	ZNF22
CDKN2B	HIST1H1C	MMP14	RUNX3	ZNF438
CETN3	HIST1H1D	MMP2	S100A11	
CFLAR	HIST1H1E	MMP9	S100Z	
CIITA	HIST1H2AC	MPEG1	S1PR2	
